# Engaging parents using web-based feedback on child growth to reduce childhood obesity: a mixed methods study

**DOI:** 10.1186/s12889-019-6618-3

**Published:** 2019-03-13

**Authors:** Rinita Dam, Heather Anne Robinson, Sarah Vince-Cain, Gill Heaton, Adam Greenstein, Matthew Sperrin, Lamiece Hassan

**Affiliations:** 1 Radcliffe Department of Medicine, Medical Sciences Division, John Radcliffe Hospital, University of Oxford, Oxford, OX3 9DU UK; 2 Division of Informatics, Imaging and Data Sciences, Faculty of Biology, Medicine and Health, The University of Manchester, Manchester, UK; 3 Manchester University NHS Foundation Trust, Manchester, UK; 4 Division of Cardiovascular Sciences, Faculty of Biology, Medicine and Health, The University of Manchester, Manchester, UK

**Keywords:** Paediatric obesity, Digital intervention, Parents, Body mass index, Mixed methods study, Health education

## Abstract

**Background:**

To measure trends in child growth and combat rising levels of obesity, Manchester University NHS Foundation Trust and the University of Manchester have developed Children’s Health and Monitoring Programme (CHAMP). CHAMP collects an annual measurement for primary school children (aged 4 to 11) in Manchester, England, and offers feedback of Body Mass Index (BMI) results to parents via a secure website. No similar digital tool exists that both provides high resolution data on the trajectory of child growth and acts as a feedback and monitoring system. This study investigates how effectively this intervention engaged with parents and supported the reduction of childhood obesity.

**Methods:**

Anonymised CHAMP registration and BMI data (UK1990) were collected between September 2013 and March 2017 from a total of 63,337 children. BMI change over time was compared in matched cohorts of 24,551 children, whose parents had and had not registered with the CHAMP website. Qualitative focus groups and interviews were used to explore perspectives among 29 key informants (parents, school and healthcare professionals) from six schools in Manchester.

**Results:**

Overweight children whose parents had not registered with the CHAMP website gained a median of 0.14 BMI centile between measurements, whilst children of CHAMP-registered parents reduced their BMI by a median of 0.4 centile per year (*P* = 0.02). Normal weight children of registered parents decreased their BMI by 0.3 centile each year, whilst those not registered increased their BMI by 0.8 centile per year (*P* = 0.001). There was no significant association between registration and BMI centile change in children already classified as obese (*P* = 0.34). A qualitative, thematic analysis revealed that the annual measurement programme was widely supported by parents and staff. A range of psychological and behavioural impacts on families were reported as a result of the monitoring and feedback processes, in some cases prompting reflection and monitoring of health and lifestyle choices.

**Conclusion:**

These early findings indicate that CHAMP, as both a monitoring system and a digital intervention, could encourage positive lifestyle change and support healthier child growth trajectories.

**Electronic supplementary material:**

The online version of this article (10.1186/s12889-019-6618-3) contains supplementary material, which is available to authorized users.

## Background

Childhood obesity is an independent risk factor for continued obesity [1], and consequently a range of health conditions in later life including cardiovascular disease [2], hypertension [3], diabetes [4, 5] and metabolic syndrome [5, 6]. Furthermore, it can affect health and wellbeing long into adulthood [7, 8]. The continuing high rates of childhood obesity in the majority of high income countries worldwide therefore pose a serious public health challenge [9, 10]. Indeed, the World Health Organisation’s (WHO) global strategy on diet, physical activity and health has emphasised the importance of preventing childhood obesity as a vital component within plans to address rising levels of non-communicable diseases [11].

Since 2006, the National Child Measurement Programme (NCMP) in England has gathered population-level data on child growth in order to monitor national population trends in childhood obesity and to inform local planning and delivery of public health initiatives [12]. Height and weight of children is routinely measured in primary schools at school entry in reception (aged 4–5) and again in their final year (Year 6, aged 10–11). Little is known about how children grow during the 5 years between these two routine measurements. Furthermore, there is variation in terms of how results are communicated and used amongst health professionals, schools and families [12].

Manchester, has one of the highest prevalence of childhood obesity in the U.K. [13]. In an effort to combat this, in 2013 Manchester University NHS Foundation Trust, working in collaboration with the University of Manchester, developed a novel annual monitoring programme called the Children’s Health and Monitoring Programme (CHAMP). The programme includes two distinct components: (a) receiving annual heights and weights for all children attending primary schools in Manchester (age 4 to 11 years), which incorporates and supplements NCMP measurements and is linked to the hospital records; and (b) a feedback system to convey BMI results for individual children directly to parents via a secure website, alongside links to healthy lifestyle advice (https://champ.mft.nhs.uk/). Thus, CHAMP is both a monitoring system and a digital intervention, directed at parents.

A recent systematic review and meta-analysis of 55 evaluation studies of parent-only interventions to reduce child obesity [14] indicated that prevention programmes have broadly positive impacts on children aged between 6 and 12 years. Nonetheless, this review identified no studies of interventions similar to CHAMP, which combined annual measurements with the delivery of BMI results to parents via a website. The aim of this study was to investigate how effectively CHAMP engaged parents and supported reductions in childhood obesity, with a view to building an evidence base for parent-only, online feedback interventions.

Research questions: which families were more likely to engage with a) the annual weighing and measuring programme and b) the CHAMP website to receive online feedback about BMI results; what were their reasons for doing so (or not); what did key stakeholders think of CHAMP; and what was the impact of CHAMP on families, including child BMI?

## Methods

### Study design

This study combined statistical analysis of CHAMP height, weight and registration data with focus groups and interviews. The study used Ellis and Hogard’s [15] three-pronged “trident” model as a theoretical framework. The prongs represent the emergence of outcomes, processes and multiple stakeholder perspectives relevant to understanding CHAMP. Quantitative and qualitative data collection took place in parallel and the two forms of data were analysed separately and combined in the reporting [16].

### Setting

This study was conducted in urban schools in Manchester, northern England. Compared with the UK as a whole, Manchester has high levels of deprivation [17], typical ethnic diversity and high prevalence of child obesity [18].

### Quantitative study

#### Data

School Health Assistants, employed by the NHS and trained by CHAMP team members from Manchester University NHS Foundation Trust (MFT), measured heights and weights using a stadiometer and calibrated Class III weighing scales connected wirelessly to an online database of children. Children were weighed and measured with their clothes on and shoes off, following NCMP operational guidelines [19]. The process was enhanced by a digital system, CHAMP Assessment Tool, which supports efficient and accurate data capture, integrated with the CHAMP parental feedback portal. Individual BMI centiles were derived based on the gender and age (to the nearest month) of the child using the UK1990 child growth standards [20, 21], via the LMSGrowth tool [22]; measurements were categorised as underweight (< 2.275), normal weight (2.275–90.789), overweight (≥90.879), obese (≥97.725), and severely obese (≥99.617) following WHO guidelines [23].

The NHS School Health Service undertake the measurements and have access to individual measurements. Parents receive personalised data information relating to their own children. However, the school leadership and staff do not have access to individual measurements. The research team at The University of Manchester received anonymised data in order to conduct quantitative analysis. Parents had the option to opt their children out of the process prior to weighing and measuring in each year of the programme.

The anonymised records of 63,337 children measured as part of CHAMP and/or NCMP during visits to 137 primary schools in Manchester between September 2013 and March 2017 were cleaned following NCMP guidelines for clinically out of range values (height, weight or BMI ±8SD from UK90 median). In total, 323 records were removed based on multiple, conflicting measurements recorded on the same date (> 0.05 kg weight difference/> 1 cm height difference), duplicate entries or missing height or weight values. Identified confounders of the relationship between CHAMP registration and change in BMI were: deprivation, as determined using the Index of Multiple Deprivation (IMD) quintile of the child’s home postcode at their most recent measurement; ethnicity, summarised as white, mixed black/white, black/British black, Asian/British Asian, ‘other’, and ‘not stated’ [24]; age; and sex. One thousand five hundred eighty-nine records lacked IMD data, and 2780 lacked ethnicity. Missing IMD/ethnicity were treated as independent categories within our model, and no missing data was imputed.

Of the 63,337 children, not all children will have been equally eligible to record measurements throughout the study time period. The expansion of CHAMP from delivery of NCMP measurements to measurement and information delivery for all primary school years between 2013 and 2015, and expansion to include 137 schools by 2015 means that more children became eligible for measurement from 2015 onwards. Finally, due to the restriction of the programme to children of primary school age, children entering Year 6 in 2013 and children entering Reception in 2017 will have been ineligible to record multiple measurements within the considered time frame (2013–2017).

#### Analysis

Cleaned data from 63,337 children were analysed. CHAMP registration status, this study’s primary exposure, was determined using an indicator of whether a child’s parent had ever registered with the CHAMP website. The first recorded BMI centile per child per academic year was used where multiple measures were recorded. The demographics of children whose parents had ever registered with the CHAMP website were compared with those whose parents had not, using t-tests for means of continuous variables, Mann-Whitney-Wilcoxon tests for medians and Chi-Squared tests for categorical variables.

To determine any independent association between CHAMP website registration and change in child BMI centile, a matched case analysis was conducted. Each child of a registered parent was matched with two unregistered children by deprivation level, ethnicity, starting BMI centile, age, and sex. Within each of three BMI centile category groups: normal, overweight, and obese, comparisons were made in median centile movement per year in registered and unregistered children via a t-test. Annual rates of BMI centile change were calculated based on the differences between individual children’s first and second calculated BMI centile (a median of 397 days apart). Few underweight children were represented in the dataset (*n* = 910), therefore we were unable to conduct a robust matched case analysis within this group.

All analyses were conducted in R, version 3.3.1 [25], using the Matchit package for the matched case analysis [26].

### Qualitative study

#### Sample

Focus group and interview participants were drawn from six primary schools in Manchester, selected to cover the range of prevalence of deprivation, BMI category and CHAMP website registration. Purposive sampling strategies were used to recruit from two broad groups: (a) parents/carers (*n* = 18); and (b) healthcare and school staff (*n* = 11). A group of parents at a local school that did not take part in the qualitative element of the study provided public involvement input into study design including recruitment strategies, participant information and dissemination strategies.

A summary of participants recruited is provided in Table 1. Parents were eligible to participate if they were residents of Manchester, aged 16 or over, English-speaking and had children who were eligible to be measured during the 2016/2017 school year. Family liaison workers working within the schools were guided to recruit a diverse sample of parents with respect to demographics (including gender, ethnicity and family size) and prior awareness of the CHAMP website. Maximum variation sampling and snowball sampling techniques were used to identify participants with relevant experiences and ensure a sufficiently diverse sample [27].

**Table 1:** Demographics of the sample for the qualitative study

(a) Parents	*n* (range)	(b) Staff	*n*
*Gender*		*Role*	
Male	2	Head/acting Head of school	4
Female	16	School Health Assistants	4
		Other school staff	1
*Age*		NHS/ public health manager	2
Mean (years)	34.1 (26–44)		
*No. of children*			
Mean	2.5 (1–6)		
*Ethnicity*			
White	12		
BME^a^	6		
*Had used CHAMP within last 6 months*			
Yes	9		
No	9		
Total	18	Total	11

^a^BME - Black and Minority Ethnic

Key personnel were deemed eligible to participate if they had a professional role relevant to the study including Heads of school (or acting Heads), School Health Assistants, and NHS and public health senior managers with a role relevant to commissioning and/or delivering childhood obesity related services.

### Data collection

Parents were invited to participate in focus group discussions and/or individual semi-structured interviews, depending on their preference. To increase flexibility and maximise opportunities for recruitment, we provided the participants with a choice of being interviewed in person or over the telephone. Focus groups took place in a private room at the school and typically lasted 60 min; interviews took place at the school and/or over the telephone and lasted up to 30 min. RD led the facilitation of the focus groups with LH acting as co-facilitator. RD carried out one-to-one and telephone interviews. All participants were asked to complete a short questionnaire to capture demographic information about them and their family. Topic guides were used to guide discussions, exploring topics including: views on healthy growth; views on the annual weighing and measuring programme; awareness and experiences of the CHAMP programme; and perceived impacts (see Additional file 1 Topic guide for parents). Key personnel were interviewed individually, typically at their place of work (see Additional file 2 Topic guide for staff). Topics explored included views on organisational approaches to combat childhood obesity, knowledge and experience of the CHAMP programme and ideas for future improvements. Data collection continued until saturation was reached, with interviewees providing no substantively new information. All focus groups and interviews were audio (digitally) recorded with the consent of participants, and transcribed verbatim.

### Analysis

Data were imported into NVivo software (version 11) and analysed thematically [28]. Data were coded using a priori framework developed from our existing understanding of the key issues. Coding then developed iteratively, with comparisons made between different stakeholders to further refine our understanding of key outcomes and processes. LH was the second coder who coded four transcripts (out of a total of 20 transcripts) – specifically, a transcript from one focus group discussion that took place with five parents, a transcript from a one-to-one parent semi-structured interview, a transcript from a one-to-one semi-structured interview with a Head of school and a transcript from a one-to-one semi-structured interview with an NHS/public health manager. Emerging findings were discussed at team meetings to resolve discrepancies and refine themes.

## Results

### Engagement with annual weighing and measuring

Of the 63,337 children in our sample, 45.0% (*n* = 28,530) had been measured once, 39.5% (*n* = 25,024) had measurements spanning two academic years and 15.4% (*n* = 9783) had measurements taken for three or more academic years between 2013/14 and 2016/17. Although, we do not have an exact number of children opting out in each year, children opted out of the programme and therefore not measured, totalled less than 1% of children eligible for the programme each year. Most parents interviewed (16 out of 18) stated that they were aware of the measuring and weighing process. The two parents (out of 18 parents that were interviewed) who were unaware that their children had been weighed and measured, stated they had not received a letter sent via satchel by the school. All 18 parents, once made aware, approved of the weighing and measuring process in principle and agreed that it was an important way to monitor children’s growth.

“*It’s good to know where you stand, where you can… If there’s a problem, you can sort it out, instead of just sitting without knowing anything*”. (Parent 5, School A).

“*Well, you at least know your kid’s healthy and understand the weight and everything like that. I think it’s a good idea to do that.”* (Parent 3, School B).

Three parents who had concerns about their children being underweight were particularly supportive of the weighing and measuring programme.

“*I was actually happy when I received a letter saying that the children were going to get weighed and measured, because like I said, I had a few concerns about my youngest child’s weight. I was appreciative of CHAMP that they did that.*” (Parent 2, School F).

Staff of the schools that agreed to take part in the study, NHS and public health staff interviewed were all supportive of the weighing and measuring process and the potential insights to be gained from the data generated. One Head of School asserted:

*“I think it’s a really good idea…I think it gives parents the information that they need to perhaps question what they’re doing…if you log on and you can see that your child is not in the green this gives you the opportunity to think. Whereas, if they’re not weighed and measured or if they’re only weighed and measured in reception and then not again until year six, then you can’t see how that is developing into a childhood obesity problem”* (Head, School B).

### Engagement with the CHAMP website

The proportion of eligible parents who had registered with the CHAMP website was 27.7% in 2015/2016 and 34.4% in 2016/2017. Children whose parents had registered with the CHAMP website were typically younger at first measurement (mean difference 4.08 months, t test *P* < 0.001), and were more likely to have been measured more than once (t test P < 0.001, Table 2). Registration was less common in children belonging to an ethnic minority (ratio = 0.74, χ^2^, *P* < 0.001), with the exception of children from mixed black and white ethnic backgrounds. Registration was lower in children from more deprived areas (χ^2^, *P* < 0.001).

**Table 2:** Comparison of children by website registration status

Variable	Description	Ever registered	Never registered	*p*-value
Participants	All children	13,536	49,801	
Male	% (n)	50.36 (6817)	50.68 (25218)	0.576
Age at start (mean)	years (SD)	7.30 (2.55)	7.64 (2.54)	<0.001
Age at start (median)	years (IQR)	7.25 (4.83–9.99)	7.83 (4.99–10.24)	<0.001
Deprivation	% (n)			<0.001
(IMD rank quintile)	Q1 (most deprived)	66.94 (9061)	77.84 (38765)	
	Q2	16.04 (2171)	12.04 (5997)	
	Q3	9.53 (1290)	5.18 (2580)	
	Q4	4.93 (668)	2.09 (1039)	
	Q5	1.54 (209)	0.62 (311)	
	Not known	1.01 (137)	2.23 (1109)	
Ethnicity	% (n)			<0.001
	White	50.43 (6826)	32.69 (16281)	
	Mixed black/white	3.66 (496)	3.42 (1703)	
	Black/British black	7.29 (987)	12.84 (6396)	
	Asian/British Asian	15.92 (2155)	19.51 (9718)	
	Other	5.07 (687)	5.70 (2838)	
	Not stated/unknown	16.98 (2298)	24.11 (12005)	
First recorded BMI	categorical, % (n)			<0.001
	Underweight	1.30 (176)	1.47 (734)	
	Normal weight	74.99 (10151)	73.65 (36680)	
	Overweight	12.50 (1692)	12.40 (6173)	
	Obese	7.28 (985)	7.82 (3892)	
	Severely obese	3.93 (532)	4.66 (2322)	
	Overweight or obese	23.71 (3209)	24.87 (12387)	
Median first recorded BMI^2^	kg/m^2^ (continuous, IQR)	16.59 (15.43–18.48)	16.71 (15.48–18.87)	0.966
Mean annual change in UK90 BMI centile^1^	centiles per annum (SD)	−0.47 (±12.14)	0.51 (±12.21)	<0.001
Median annual change in UK90 BMI centile^1^	centiles per annum (IQR)	− 0.14 (−6.01–4.44)	0.07 (−4.88–5.33)	<0.001
Median time span of recorded BMI values^1^	days (IQR)	396 (365–427)	397 (366–427)	<0.001
Total number of BMI measurements recorded 2013–2017		26,982	81,113	<0.001
Mean number of BMI measurements recorded per child 2013–2017		1.99	1.63	<0.001

^2^Based on values from 34,803 children with multiple recorded measurements 2013/14–2016/17

The median initial BMI centile of children of CHAMP-registered parents was lower than that of children with unregistered parents (16.59 versus 16.74; Wilcox P < 0.001); the registered group included higher proportions of normal and overweight children, and fewer underweight, obese and severely obese children (χ^2^, P < 0.001). The two groups included similar proportions of boys and girls (χ^2^, *P* = 0.576, Table 2).

We note a current selection bias in parents who successfully interact with CHAMP. Children whose parents had registered with CHAMP were generally younger (LM, *P* < 2e-16), and from more affluent wards in Manchester (P < 2e-16). They were less likely to belong to an ethnic minority (P < 2e-16). Children of Caribbean (effect size = − 0.177), ‘other black’ (−1.633) and Bangladeshi ethnicities (− 0.168) were the least likely to participate. There was no gender difference in registration frequency between registered and unregistered children (LM, *P* = 0.7).

Interviews revealed that the process by which parents became aware of, and subsequently accessed the CHAMP website was inconsistent. Among the parents who had prior knowledge of the CHAMP website, the majority had previously logged in to view their children’s results. However, only a few parents could clearly recall the process of receiving a letter from the school encouraging them to register with the CHAMP website and a subsequent letter providing login details.

The main reason reported for not accessing CHAMP was not receiving the letter with instructions prompting parents to register and/or log into CHAMP. A few parents reported difficulties registering, logging in and/or retrieving results. Other factors cited included lack of time and misplacing the registration instructions. School staff interviewed identified a number of reasons why parents may have not been aware of the CHAMP website. These included satchel post getting mislaid and difficulties with understanding written English (especially among those for whom English is not their first language). As one Head of School commented:

“*We did hand them out to the children so there’s the risk then they do get mislaid. But also within the makeup of our school, there are some families who have English as a second language and so they sometimes struggle with those forms of communication and language barriers … [plus] some people may not be fluent readers in their first language.*” (Head, School C).

### Associations between CHAMP registration and changes in child BMI

Table 3 describes the BMI centile movements of 24, 551 matched children between their first and second recorded measurements (a median of 397 days apart). Annual numbers of total participants have been added to the supplementary data (see Additional file 3 Table Annual numbers of total participants). Children of CHAMP-registered parents with normal starting BMIs were more likely to remain within the normal BMI range than children of unregistered parents (χ^2^, *P* < 0.01), and showed significant differences in median annual centile change (t test, *P* < 0.001). Within the normal weight category, children in both groups were similarly likely to transition towards the BMI median (50th UK90 centile) between measurements (χ^2^, *P* = 0.347), suggesting that the two groups differed in the size of the observed BMI centile difference, rather than in the direction of movement of individuals.

**Table 3:** Summary of UK90 BMI centile movement of children paired in matched case analysis between first and second assessments

BMI category (child)	Website registered (parent)	n	Median annual centile change^3^	% moving towards UK90 50th centile	% constant centile	% moving away from UK90 50th centile
Normal weight	Yes	6016	−0.68***	44.08	0.05	55.87
	No	12,890	+ 0.30	43.69	0.02	56.30
Over weight	Yes	893	−0.40*	53.75*	0.00	46.25
	No	2126	+ 0.14	48.02	0.05	51.93
Obese	Yes	700	−0.02	53.57	4.14	42.29
	No	1926	<0.01	49.74	4.98	45.28

^3^*P < 0.05, **P < 0.01,***P < 0.001

Overweight children who had not registered with CHAMP gained a median of 0.14 BMI centiles between measurements, whilst those whose parents had registered decreased their BMI by a median of 0.4 centiles per year (t test, *P* = 0.014). Additionally, overweight children were more likely to move towards the BMI median (50th Centile) between measurements if their parents had registered with CHAMP (χ^2^, P = 0.014), suggesting that the trajectories of individuals differed.

For a 10-year old boy aged 120 months, in the middle of the overweight category (95th UK90 percentile), and of median height for this dataset (140.5 cm):

- Not registered with CHAMP (gains 0.14 BMI percentile to 95.14th percentile) corresponds to 0.18 kg gain.
- Registered with CHAMP (drops 0.4 BMI percentile to 94.6th percentile) corresponds to 0.48 kg loss.

Obese children showed minimal movement in BMI centile, regardless of whether their parents were registered with the website (t test, *P* = 0.255), and children in each group were similarly likely to move towards or away from normal values between measurements (χ^2^, *P* = 0.105).

Within the obese and overweight matched BMI categories, boys and girls showed similar overall annual centile movements (t tests, *P* = 0.98, *P* = 0.79). However, girls in the normal weight category showed a greater mean annual BMI centile increase than boys (ANOVA, *P* = 0.02), possibly due to gender differences in growth timing. This relationship was consistent regardless of registration status (ANOVA, *P* = 0.62).

Parents readily described a range of measures that they were using to support their children to be healthy, including making healthy meals, avoiding unhealthy food and encouraging their children to be physically active. Most of the parents we spoke to believed they already encouraged healthy lifestyles among their children. Whilst some did express frustrations and challenges (for example children being ‘fussy eaters’), they felt they were already doing their best, regardless of the weighing and measuring results.

“*I guess that’s my confidence in myself. You just wouldn’t want parents doing really silly things, like if they had an underweight result and they’re force feeding their children or if they start starving their kids*.” (Parent 1, School B).

“*So I’m doing exactly what we need to do, just her weight – there’s nothing I can do because her bone weight is heavy*.” (Parent 1, School C).

A few parents specifically reported having made further lifestyle changes within their family to improve their health directly in response to being weighed and measured at school or in response to feedback about their children’s BMI results.

“*Last year when my daughter was weighed in school we were told she was overweight… It was a real concern for the school, they were monitoring the weight, thinking of activities that she could do. It was stressful for us, so that’s when we started looking into it*.” (Parent 3, School C).

### Psychological impacts of CHAMP on families

Qualitative analysis yielded three subthemes, indicating a range of psychological impacts of CHAMP: a judgement of parenting; increased reflection and monitoring and psychological impacts on children (Table 4).

**Table 4:** Psychological impacts of CHAMP on families

Subtheme	Description	Example quote
A judgement of parenting	Parents may feel judged or reassured, depending on the BMI result	“*If your child is not where they should be, it can give you feelings that you are failing as a parent*.”
Increased reflection and monitoring	CHAMP can prompt parents to stop, reflect and monitor	“*I have to check whether she is eating well, whether she is growing, but I have to look after her weight*.”
Psychological impacts on children	Concern about children worrying over weight from a young age	“*I think [she’s] young to be worrying about weight.”*

### A judgement of parenting

Whilst parents felt that children ought to be weighed and measured, qualitative analysis suggested that discussing issues surrounding healthy growth remained an emotionally loaded conversation, especially where there were already concerns about their children’s weight.

Reactions varied depending on the parent, the child and the BMI result. For some parents, the process was routine and provided additional (if unsurprising) reassurance that the child was growing as expected. A few parents reported a sense of relief when results indicated their children’s results were within the normal range. Conversely, if children did not fall within the healthy weight range for their age, some parents reported to ‘feeling judged’. Parents who articulated such feelings spoke of the results being taken as a reflection of their parenting abilities.

“*Your child is a reflection of you, so if you’re being told that your child is overweight, some people could take that quite personally as a reflection of themselves*.” (Parent 1, School B).

“*As a parent, if there’s something that you feel like that you are not doing, if your child is not where they should be, it can give you feelings that you are failing as a parent*.” (Parent 1, School F).

School staff acknowledged that raising issues with the parents about their children’s eating habits could be contentious and emphasised the importance in building and maintaining relationships with parents to engender trust and subsequently, discuss weight concerns with the school staff.

“*It’s very tentative isn’t it, because you don’t want to offend people, you want to have influence with them; and you can only do that gently, gently by building a relationship, you can’t do that in a forceful way, because you’re just going against what you’re trying to achieve then*.” (Acting Head, School A).

### Increased reflection and monitoring

Regardless of the actual outcome, the weighing and measuring process could encourage parents to pause, reflect and take a closer look at their child’s growth. For some parents, it was results that were outside the normal range that caused them to reflect on the results most.

“When I got [her’s], my middle one, she came back as slightly underweight, in a way that made me look at it more.” (Parent 1, School B).

“It is a concern that I have to follow her growth. I have to check whether she is eating well, whether she is growing, but I have to look after her weight.” (Parent 1, School E).

Nonetheless, two parents of children who were within the normal range also spoke of continuing to use the CHAMP website to “keep an eye” on their children’s growth in future.

### Psychological impacts on children

Parents expressed some concerns about the potential psychological impacts of sharing the weighing and measuring results with their children. Some parents reported that if children felt ‘labelled’ as overweight from a young age, this could result in negative psychological impacts. Parents themselves often preferred to use more euphemistic terms to describe weight and body types of their own and others’ children, such as, “chubby”, “puppy fat” and “solid”.

“*So, [she], my youngest came back in the overweight bit, and I don’t like the idea of her being labelled as that, because I think I’m feeding her quite well…I don’t know, what other term would you use apart from overweight*?” (Parent 1, School C).

Parents generally did not choose to share results with their children. The weighing and measuring process sometimes inadvertently prompted sensitive conversations between parents and children. However, there appeared to be a broad consensus among parents that, unless the problem was particularly severe, primary school aged children were too young to worry about their weight.

“*She’s only nine, nearly ten at the end of the day. I think it’s young to be worrying about weight because she’s going through puberty now. So her body is going to be changing*.” (Parent 2, School A).

## Discussion

CHAMP website registration appears to associate with improved BMI centile outcomes in primary school children. Overweight children of CHAMP-registered parents were more likely to decrease their BMI centile and therefore to move towards or into the normal weight range than overweight children of parents who were not registered with CHAMP. Additionally, children of CHAMP-registered parents who started within the normal weight range were more likely to move towards the 50th UK90 BMI centile or to maintain their existing centile than normal weight children of parents who were not registered with CHAMP. Both normal weight and overweight children who had not registered with CHAMP typically increased centile between measurements, whilst those who had registered reversed this trend. Neither of these relationships were observed in children already classified as obese, who in both groups increased BMI centile over time.

Qualitative analysis revealed that whilst parents and staff felt that children ought to be weighed and measured regularly, communicating issues with parents about healthy and unhealthy growth remained an emotionally loaded conversation with potential for psychological, as well as behavioural impacts.

These findings build on the findings of two major systematic reviews related to childhood obesity prevention. A recent systematic review concluded that childhood obesity prevention programmes have broadly had positive impacts on children between the ages of 6 and 12 years, but that not all children involved in such programmes are impacted equally [14]. Furthermore a second review [29] of interventions targeted towards parents found limited evidence that these interventions helped to reduce BMI centile in children. Nonetheless, neither identified any reports of school-wide interventions similar to CHAMP, which delivered regular annual BMI measurements to parents via digital methods.

Findings from the qualitative analysis suggested that parents and staff were supportive in principle of annual measuring and feedback. Though registrations grew year on year reaching 33% in 2016/17, this still leaves families of two-thirds of children who had been measured, but had not registered with the CHAMP website in order to view the results online. Given that Manchester has high levels of deprivation, it is likely that some parents may require support in becoming digitally connected consumers of healthcare related information. Where support has been offered, we have seen individual schools increase their numbers of registered parents considerably.

Children of parents who had registered with CHAMP were slightly but not significantly less likely to be overweight, underweight or obese (*P* > 0.05). Interviews with parents, however, revealed they were unaware that the CHAMP team intended that they register and check results on an annual basis, which is the main premise of CHAMP and a unique element of the programme. Therefore, we propose that this lack of awareness constitutes the primary barrier to registration. Although the CHAMP system cannot communicate with a parent until the parent has registered via the website, additional measures such as text messages are being introduced to prompt repeat engagement among those that have registered.

CHAMP appeared to associate with improved BMI centile outcomes for overweight and normal weight primary school children, but not obese children. A BMI centile reduction does not necessarily equate to weight loss; this could occur when the rate of weight increase is lower than the rate of height increase or where weight is periodically stable alongside height increase. In Manchester, we know that children are tracking upwards across BMI centiles between the ages of 4 and 11 years. We found that 56% of children with normal starting weights moved away from the 50th centile within their first year of registration, typically by increasing their BMI centile. The lack of significant differences between registered and non-registered groups shows only that weight gain was not typically reversed in this period. It is possible that obese children may need different interventions. Nonetheless, we regard disrupting the rate of BMI centile gain among normal and overweight children using CHAMP as a positive outcome.

The findings in this study highlight difficulties in communicating issues about healthy and unhealthy growth to parents. Interviewee responses revealed that some parents viewed their children’s BMI results as a reflection on their own parental skills. Consequently, if children did not fall within the normal BMI range, parents could be left ‘feeling judged’. The wider literature has reported mixed parental responses to messages about child weighing and measuring results. Whilst some published evaluations of interventions delivering BMI results to parents via traditional report card or letter media have demonstrated positive outcomes in terms of weight loss [30, 31] and improving the accuracy of weight perceptions [32], some have noted reactions of dissatisfaction and even disgust [12, 33, 34]. It is common finding in the wider literature that health promotion messages may be perceived as threats and greeted with ‘defensive’ reactions; depending on the particular mechanisms at work, these may or may act as barriers to accepting health-promoting information [35]. Indeed, in one study the authors noted that some parents who were told that their children were overweight did, following the initial shock, eventually express an intention to seek help [34].

In considering the impact of BMI messages, some have noted concerns that identifying a child as overweight could potentially lead to negative effects on body image [36]. CHAMP guards against this by following the same principles as the NCMP in England; BMI results are communicated directly to parents rather than the child, with the additional benefit that in CHAMP online feedback enables them to view the results privately at a convenient time and place. Research on the NCMP has shown that parents want feedback on their children’s BMI and has reported no adverse effects on self-esteem and weight-related teasing in overweight and obese children [37, 38]. More widely, a systematic review of the evidence related to screening and treatment for obesity on children and adolescents found no evidence of these programs causing harm [39]. We have no reason to believe this would not also apply to CHAMP. On balance, whilst there is the possibility of BMI feedback having a negative emotional impact, we believe this risk to be low in this particular age group and outweighed by the potential benefits. Indeed, the collation of more regular measurements via CHAMP could support the identification of concerning underweight growth trajectories, helping to actively identify children with potential eating disorders.

### Strengths and limitations

To our knowledge, this is the first study to focus on a digital intervention that aims to engage parents in supporting healthy child growth on an annual basis using a digital feedback intervention. The study benefited from a mixed methods approach which integrated longitudinal data about child growth trajectories and outcomes with rich, qualitative data to yield insights into both the intervention’s outcomes and mechanisms. Participation in annual weighing and measuring among children was extremely high (over 99%) yielding a large sample of children from Manchester.

Nonetheless, there were a number of limitations. Whilst statistical analyses indicated a number of significant associations with CHAMP registration, unlike a randomised controlled trial (RCT), the observational design of the study does not allow us to discern which parts of the programme were responsible for these effects or indeed whether these were wholly due to the programme. An RCT design would have been scientifically preferable, but was not practically possible for a number of reasons. First, given that CHAMP was introduced to Manchester in 2013, the programme is now embedded within Manchester and there would be a reluctance to withdraw current practice. Second, there was insufficient resource to support a sufficiently large, multi-site RCT required to yield meaningful results. The current study provided a timely opportunity to quickly generate robust preliminary results in a smaller sample in preparation for a future RCT.

Multiple measurements, required to measure BMI change, were not available for all children for a number of reasons. Parents opting out of the programme, along with unscheduled absences, did result in a minority of children not being weighed and measured each year, which will have had a small impact. Of greater significance is the fact that CHAMP was progressively rolled out over 2013–2015, going from only measuring at Reception and Year 6 (as part of the NCMP) to a yearly programme of measurement by 2015. In addition, some children will only have a single measurement due to the timing of their entry or departure from primary school (e.g. if they were measured in reception in 2017 or in Year 6 in 2013. Furthermore, children in private education in Manchester (21 schools) do not participate in the NCMP and were not offered CHAMP, and demographic data was not available regarding the small percentage of children who had opted out of the programme.

Matching the registered and unregistered groups on key demographic variables acted as our primary means of addressing selection bias statistically, maximising their comparability. A limitation of this method is that not all children could be matched to a similar child, meaning fewer children were used in the matched case analysis than had multiple measurements recorded (24,551 out of 25,024). We believe this was the most robust approach under the circumstances. Nonetheless, we do note a potential for selection bias in the over-representation of children from ‘early adopter’ schools who implemented CHAMP from 2013; the analysis necessarily assumes their selection to be random. Furthermore, the latest point at which children could be measured was on entry to Year 6, meaning we could not infer potential for BMI change beyond this point.

Recruitment to the qualitative study was challenging for a number of reasons, including the challenges of engaging parents about a sensitive topic, the researchers’ limited ability to access schools and difficulties engaging working parents. Nonetheless, whilst findings from our qualitative study are limited by the small sample size, which was mostly female, they are nonetheless valuable, as the views of the parents on this subject are often difficult to obtain. Parents were not directly asked to disclose their own or their children’s weight or measurements, therefore we could not account for this in the sample selection or analysis. This was a deliberate decision made a priori on an ethical basis. During the course of conducting interviews with the parents, potential social desirability response bias may have occurred when using both face-to-face and telephone interview techniques; parents may have wanted to please interviewers or portray themselves as ‘good’ parents which could have modified their responses. However, when it came to analysing responses from the two interview techniques, we found the same themes emerging.

Finally, due to the programme being established relatively recently, few children had more than three measurements and thus we were only able to follow up children over limited time periods. We also lacked sufficient power to assess the impact of CHAMP for underweight children, which represented fewer than 2% of all children measured. These problems are likely to be overcome as time progresses and the programme continues to collect data.

### Implications for future research and policy

The results of this study demonstrate the potential of CHAMP as both a monitoring system and feedback intervention. Measuring the effectiveness of child obesity interventions may be leveraged by using large longitudinal datasets from children [40]. CHAMP provides higher resolution data on the trajectory of child growth than currently available from the NCMP, acting as a robust monitoring system and a reference for evaluating future interventions. Furthermore, CHAMP provides the basis of a simple, scalable feedback intervention aimed at engaging parents to prevent and/or reduce childhood obesity. To maximise value, CHAMP needs to be rigorously evaluated, through an RCT, to investigate its value and scalability. This should include proper assessment of the potential benefits and risks arising from screening in this way.

The findings highlight the complex psychological and behavioural factors at work when communicating sensitive messages about child growth. Concerns have previously been raised that the NCMP’s weighing and measuring programme had limited impact on parental perceptions and did not adequately link to follow on support for children identified as overweight or obese [41]. CHAMP, and programmes like it, that rely on digital mediums are not immune to such criticism and thus further work will be required to optimise engagement, craft effective risk communication messages that effectively overcome psychological barriers and help schools and health professionals (‘the wider system’) to provide appropriate follow-on support to families.

## Conclusions

CHAMP, a programme which combined annual height and weight measurements of children with web-based BMI feedback to parents, appeared to associate with improved BMI centile outcomes for overweight and normal weight primary school children. Whilst parents and staff were supportive of annual measuring and feedback in principle, communicating issues about healthy and unhealthy growth remained an emotionally loaded conversation with potential for psychological, as well as behavioural, impacts. CHAMP needs to be rigorously evaluated through a randomised controlled trial to investigate the full range of health, behavioural and psychological impacts and to determine its value and scalability.

## Additional file

Additional file 1: Topic guide for parents. Parent/carer focus group/interview guide: CHAMP and non-CHAMP users. (DOC 103 kb)

Additional file 2: Topic guide for staff. Staff interview topic guide. (DOC 105 kb)

Additional file 3: Table. Annual numbers of total participants. (DOC 28 kb)

## Abbreviations

BMI: Body Mass Index
CDC: Centers for Disease Control and Prevention
CHAMP: Children’s Health and Monitoring Programme
IMD: Index of Multiple Derivation
MFT: Manchester University NHS Foundation Trust
NCMP: National Child Measurement Programme
OWOB: Overweight or obese
RCOM: Reducing Childhood Obesity in Manchester
RCPCH: Royal College of Paediatric and Child Health
RCT: Randomised Controlled Trial
UK90: World Health Organisation (WHO) Child Growth Standards [21]
WHO: World Health Organisation
