# Supplementary material for: Engaging parents using web-based feedback on child growth to reduce childhood obesity: a mixed methods study
Source: BMC Public Health. 2019 Mar 13;19:300. doi: 10.1186/s12889-019-6618-3 (PMC6415344; doi:10.1186/s12889-019-6618-3)
Supplement: Supplementary file 1 — Topic guide for parents. Parent/carer focus group/interview guide: CHAMP and non-CHAMP users. (DOC 103 kb) [file 12889_2019_6618_MOESM1_ESM.doc]

**Evaluation of the Children’s Health and Monitoring Programme (CHAMP) in raising parental awareness of childhood obesity in Manchester**

**Parent/carer focus group/interview guide: CHAMP and non-CHAMP users**

1. How do you know your child is growing appropriately as they should?
2. Did you know that your child was measured this year in school?

*Prompt*: Did you get any letters/text messages from the school about this?

1. Have you heard of Children’s Health and Monitoring Programme (CHAMP)? What do you think CHAMP is? Who does it include?
2. For this year’s measuring programme that your child was part of, what did you think and how did it make you feel when you received a letter from CHAMP asking you to take part?
3. What did you think of this year’s measuring process?

*Prompt*: Strengths or weaknesses. Is there anything you would have liked for them to have done differently?

1. After your child was measured, did you find out your child’s results? If so, how?

*Prompt*: In what format was the feedback provided?

*Prompt:* Did you register on to the CHAMP website then to view your child’s measurement results? When did you register on the CHAMP website?

*Prompt:* If not, how did you obtain your child’s measurement results? Do you have a computer and/or access to the internet?

1. What was your initial reaction when you received your child’s results? Were they as expected?
2. What did you do after receiving feedback? Is there any support that you would like?
3. If your child is in Reception or Year 6, A Better Life (ABL) may contact you if the results from your child’s measurements indicate that he/she is above healthy weight. This is indicated in your invitation letter. How did you feel about being asked to share your contact details and your child’s data with ABL?
4. ***For only those who registered on to CHAMP*:** If you have access to a computer and/internet, how did you feel when you saw your child’s BMI on the website? Are you happy/agree with the feedback? If so, why? If not, why not?
5. ***For only those who registered on to CHAMP:*** Did you have any difficulties in logging on to the website? If so, were they resolved, how were they resolved and by whom?
6. In an ideal world, how would you like to receive feedback about your child’s results?

*Prompt*: Online vs letter

1. ***For only those who registered on to CHAMP:*** What made you sign up to CHAMP?
2. ***For only those who did not register on to CHAMP:*** What were your main reasons for not registering on to the CHAMP website? Were there any factors that stopped you from registering on to the website?
3. ***For only those who registered on to CHAMP:*** Would you like to use the system for anything else? E.g. immunisations, hearing checks, eye tests or any other health checks?
