# Supplementary material for: Engaging parents using web-based feedback on child growth to reduce childhood obesity: a mixed methods study
Source: BMC Public Health. 2019 Mar 13;19:300. doi: 10.1186/s12889-019-6618-3 (PMC6415344; doi:10.1186/s12889-019-6618-3)
Supplement: Supplementary file 2 — Topic guide for staff. Staff interview topic guide. (DOC 105 kb) [file 12889_2019_6618_MOESM2_ESM.doc]

**Evaluation of the Children’s Health and Monitoring Programme (CHAMP) in raising parental awareness of childhood obesity in Manchester**

**Staff interview topic guide**

1. **Introduction and greeting**

- Name
- Research project overview
- Interview format
- Consent
- Confidentiality
- Audio recording
- Right to withdraw/ refuse to answer questions
- Any questions before we start?

1. **Role background**

- Can you tell me about your role here?
  - - - What is your current role and job description?
      - What is your recent job history and have you moved between organisations?

1. **Priorities related to combating childhood obesity within the organisation**

- What information do you have access to about childhood obesity?
- How high a priority is tackling childhood obesity for your organisation?
- Can you tell me about the ways in which your organisation is dealing with the issue of childhood obesity?
  - - - What would you say are the key strategies with regards to combating childhood obesity within your organisation?
      - What resources have you got or you will have in place in order to achieve this?
      - What are the key tasks for you personally in tackling childhood obesity in the role you are in?
      - What are the greatest challenges facing you and the organisation at present in terms of tackling childhood obesity? (**Probe**: organisational changes or financial deficits)

1. **Children’s Health and Monitoring Programme (CHAMP)**

- Can you tell me about how your organisation is involved with CHAMP?
  - - - What do you see as the key ingredients of the CHAMP programme?
      - What do you think of CHAMP: (i) Taking measurements of ***all*** primary school aged children every year, (ii) Inviting parents to keep abreast of child’s growth annually on line and (iii) Inviting parents viewing results on line and accessing website links for (support)?
      - What aspects of CHAMP are working well and why?
      - What aspects of CHAMP are not working so well and why? (**Probe**: How are the success/failure factors unique/different to what’s going on elsewhere?)
      - What do you see are the main barriers to CHAMP and how do you think they can be overcome?
      - What do you want to see CHAMP achieve in the fight against childhood obesity? What’s your vision for CHAMP and where does it need to go in the future to improve?
      - What part, if any, do you think you play in your role alongside CHAMP?

1. **Working with families**

- Can you tell me about whether and how you work with families in the fight against childhood obesity?
  - - - In what ways do you come into contact and work together?
      - How much of a priority is childhood obesity for the families you work with?
      - How does CHAMP fit into the work you do with families?
      - Which aspects are most challenging? Why is this the case?

1. **Inter-organisational working**

- Can you tell me about whether and how you work with other organisations that are involved in the fight against childhood obesity?
  - - - Which are the key partners or organisations that your organisation works with locally? (RCOM, Public Health England, Public Health Manchester (including Manchester local authority), CMFT, primary/secondary schools, School Health Service, charities and other private stakeholders)?
      - How do you work together? What are the channels of communication?
      - How would you characterise your inter-organisational working relationships? Which work best?
      - Which are the most challenging? Why is this the case?
      - Do you think that there is overlap or duplication of work between organisations?
      - How do you relate to national level bodies?

1. **Other**

- Reflecting on our conversation, please could you summarise the main strengths and weaknesses of CHAMP?
- Anything you expected me to ask about that we haven’t discussed or you would like to add?

**Thank you for taking part in this study.**
