# Supplementary material for: Engaging parents using web-based feedback on child growth to reduce childhood obesity: a mixed methods study
Source: BMC Public Health. 2019 Mar 13;19:300. doi: 10.1186/s12889-019-6618-3 (PMC6415344; doi:10.1186/s12889-019-6618-3)
Supplement: Supplementary file 3 — Table. Annual numbers of total participants. (DOC 28 kb) [file 12889_2019_6618_MOESM3_ESM.doc]

**Supplementary file 3 Table: Annual numbers of total participants**

| Academic Year | Total measured children |
| --- | --- |
| 2013/2014 | 10,898 |
| 2014/2015 | 12,422 |
| 2015/2016 | 42,614 |
| 2016/2017 | 42,345 |
